# Modified Embedded-Atom Interatomic Potential Parameters of the Ti–Cr Binary and Ti–Cr–N Ternary Systems

**DOI:** 10.3389/fchem.2021.773015

**Published:** 2021-10-29

**Authors:** Shoubing Ding, Yue Li, Yiying Luo, Zhimin Wu, Xinqiang Wang

**Affiliations:** ^1^ Chongqing Key Laboratory of Photoelectric Functional Materials, College of Physics and Electronic Engineering, Chongqing Normal University, Chongqing, China; ^2^ School of Physics, Chongqing University, Chongqing, China

**Keywords:** Ti-Cr-N ternary system, atomic simulation, multilayered coatings, 2NN MEAM, interaction potential PACS:31.15.bu; 62.20.dq; 68.35.-p; 71.15.Nc

## Abstract

The second nearest-neighbor modified embedded-atom method (2NN MEAM) potential parameters of the Ti–Cr binary and Ti–Cr–N ternary systems are optimized in accordance with the 2NN MEAM method. The novel constructed potential parameters can well reproduce the multiple fundamental physical characteristics of binary and ternary systems and reasonably agree with the first-principles calculation or experimental data. Thus, the newly constructed 2NN MEAM potential parameters can be used for atomic simulations to determine the underlying principle of the hardness enhancement of TiN/CrN multilayered coatings.

## Introduction

Transition metal nitride multilayer coatings are widely applied because of their excellent hardness, high thermal and chemical stability, and high oxidation and wear resistance (Chu et al., 1999; Zhou et al., 1999; Lee et al., 2005). TiN/CrN multilayered coating, a typical example that comprises alternating lamellas of its two mononitrides, exhibits much higher hardness while maintaining excellent corrosion resistance, high thermal stability, and oxidation resistance of CrN (Su et al., 2008; Luo et al., 2011; Koseki et al., 2015). Therefore, TiN/CrN multilayered coatings are promising for industrial fields such as for use in cutting tools, wherein both hardness and temperature stability are significantly important characteristics (Barshilia et al., 2003). Hence, investigating the improved chemical and mechanical characteristics of TiN/CrN multilayered coatings to clarify the underlying mechanism is fundamentally significant.

Indeed, extensive research has been conducted *via* experimental and computational simulations to identify the reason for the property improvement afforded by TiN/CrN multilayered coatings (Nordin et al., 1999; Mendibide et al., 2005; Steyer et al., 2008; Yin et al., 2012). Nordin et al. determined that the interfaces quantity in the multilayers significantly impacts the corrosion resistance of TiN/CrN multilayered coatings (Nordin et al., 1999). Moreover, Mendibide et al. discovered that the crack propagation mode shift caused by the fluctuating residual stress field can improve the wear resistance of TiN/CrN multilayered coatings (Mendibide et al., 2005; Steyer et al., 2008). Furthermore, the tensile behavior has recently been shown to be extremely important for learning the mechanical properties of multilayers since it is strongly correlative with the fracture and dislocation nucleation (Yin et al., 2012). However, sufficient information about the tensile process and ultimate geometry is difficult to experimentally obtain, particularly at the atomic level. Atomic simulations, such as the first-principles calculation on the basis of the density functional theory (DFT) and molecular dynamics (MD) simulation, afford microstructural information about the multilayered coatings and provide another useful method for investigating the physical characteristics of TiN/CrN multilayered coatings. In fact, numerous first-principles computing has been performed for transition metal nitride multilayered coatings (Stampfl and Freeman, 2012; Yin et al., 2012; Yin et al., 2014). But because of the size (or atoms quantity) constraints for constructing the supercell, DFT mainly focuses on the framework, stability, and strength of the interfaces in multilayered coatings. DFT is still difficult to apply in the investigation of a complex system’s properties, especially the evolution of defects during the tensile process.

MD simulation, where over a million atoms are considered, is a useful method for gaining insights into the deformation and evolution of multilayered coatings. However, interatomic potentials need to be selected before the physical properties of TiN/CrN multilayered coatings are studied using MD simulation. The quality of interatomic potentials significantly affects the validity and dependability of MD simulations. A good interatomic potential could correctly reproduce multiple fundamental physical characteristics of correlative material systems. Therefore, 2NN MEAM potential (Lee and Baskes, 2000; Lee et al., 2001), developed from the embedded-atom method, is thought to be highly suitable for the multilayers as it can describe various elements using the same functional formalism (Daw and Baskes, 1983; Foiles et al., 1986).

For TiN/CrN multilayered coatings, the 2NN MEAM potential parameters of the Ti–Cr–N ternary system are needed to clarify the underlying mechanism of the hardness enhancement; However, they are not yet available. The 2NN MEAM potential parameters of a multicomponent alloy system can be determined by the 2NN MEAM potential parameters of the unary and binary systems. Thus, to obtain the potential parameters of the Ti–Cr–N ternary system, the potential parameters of the unary (Ti, Cr, and N) and binary (Ti–N, Cr–N, and Ti–Cr) systems are required. The potential parameters of Ti (Kim et al., 2006), Cr (Ding and Wang, 2019), N (Baskes, 1992), Ti–N(Ding and Wang, 2019) and Cr–N(Ding and Wang, 2019) systems are already obtained. Hence, before calculating the potential parameters of the Ti–Cr–N ternary system, the parameters of the Ti–Cr binary system shall be obtained.

As a part of the long-term project investigating the underlying mechanism of the hardness enhancement of TiN/CrN multilayered coatings at the atomic level and developing interatomic potential parameters of relevant systems to enable such investigations, this study aims to develop the potential parameters of the Ti–Cr binary system and extend them to the Ti–Cr–N ternary system. The rest part is described as below. The 2NN MEAM method and how to determine the potential parameters are depicted in *Methodology*. In *Verification of the Interaction Potential*, the reliability of the newly developed potential parameters is checked by the comparison of fundamental physical characteristics of correlative materials with the available experimental data and first-principles calculation results. Finally, *Summary* presents a summary.

## Methodology

### Interaction Potential

In 2NN MEAM potential, the total energy of a multicomponent system is expressed as

$$E=\sum_{i}\left[F_{i}\left({\bar{\rho }}_{i}\right)+\frac{1}{2}\sum_{j\left(\neq i\right)}S_{ij}\phi_{ij}\left(R_{ij}\right)\right],$$ (1)

where *F*_*i*_ is the embedding function for embedding the atom *i* within a background electron density ${\bar{\rho }}_{i}$ and the pair potential $\phi_{ij}\left(R_{ij}\right)$ and screening function *S*_*ij*_ are evaluated at the distances of atoms *i*, *j*, and *R*_*ij*_. To calculate the energy, *F*_*i*_ and $\phi_{ij}\left(R_{ij}\right)$ are required.

The embedding energy *F*_*i*_ is as follows (Baskes, 1992):

$$F\left(\bar{\rho }\right)=AE_{c}\left(\frac{\bar{\rho }}{\bar{\rho^{0}}}\right)\ln\left(\frac{\bar{\rho }}{\bar{\rho^{0}}}\right),$$ (2)

where *A* is a tunable parameter, and *E*_c_ and $\bar{\rho^{0}}$ are the cohesive energy and background electron density of a reference framework, respectively. The detailed mathematical forms of 2NN MEAM can be found in literature (Baskes, 1997; Lee and Baskes, 2000; Lee et al., 2001; Kim et al., 2006) and is not repeated here. Only the major aspects of the model that determine ternary interaction potentials are concisely described in this section.

In 2NN MEAM potential, no unequivocal functional expression is assigned to the pair interaction $\phi_{ij}\left(R_{ij}\right)$ . However, a reference framework is defined, wherein each atom is sitting in the exact lattice points. The total energy per atom is achieved as a function of the nearest-neighbor distance using the state universal equation presented by Rose et al. (1984). Then, $\phi_{ij}\left(R_{ij}\right)$ is estimated by the embedding energy and the total energy per atom. The universal equation of state is

$$E^{u}\left(R\right)=-E_{c}\left(1+a^{*}+da^{*3}\right)e^{-a*},$$ (3)

Here, *d* is a tunable parameter,

$$a^{*}=\alpha \left(\frac{R}{r_{e}}-1\right),$$ (4)

and

$$\alpha ={\left(\frac{9B\Omega }{Ec}\right)}^{\frac{1}{2}}.$$ (5)

In Equations 3–5, *r*_e_, *B*, Ω and *E*_c_ represent the nearest-neighbor distance, the bulk modulus, the equilibrium atomic volume, and the cohesive energy of the equilibrium reference framework, respectively. The values of them and *d* are supposed or determined via first-principles computing or experiments.

In 2NN MEAM, the pair interaction between constituent elements needs to be confirmed to explain a multicomponent system. The total energy of a reference framework can be obtained as below:

$$E^{u}\left(R\right)=F\left(\bar{\rho^{0}}\left(R\right)\right)+\frac{Z_{1}}{2}\phi \left(R\right)+\frac{Z_{2}S}{2}\phi \left(aR\right),$$ (6)

where *Z*_1_, *Z*_2_ and *a* represent the number of first and second nearest-neighbor atoms, and the ratio between them, respectively. Additionally, *S* represents the screening factor for the 2NN interactions. For a given reference, the values of *S* and *a* are constants. Then, the pair potentials can be obtained from Eqs 3, 6:

$$\phi \left(R\right)=\psi \left(R\right)+\sum_{n=1}{\left(-1\right)}^{n}{\left(\frac{Z_{2}S}{Z_{1}}\right)}^{n}\psi \left(a^{n}R\right),$$ (7)

where

$$\psi \left(R\right)=\phi \left(R\right)+\frac{Z_{2}S}{Z_{1}}\phi \left(aR\right).$$ (8)

Here, the summation is always executed unless an accurate energy value per atom is acquired.

The many-body screening involved in MEAM (Baskes, 1997) differentiates it from other empirical potentials. The *S*_*ij*_ represents the impact of the neighbor atom *k* on the interaction between atoms *i* and *j*, which is the product of the screening factors determined by all the other neighbor atoms *k*:

$$S_{ij}=\prod_{k\neq i,j}S_{ikj}.$$ (9)

The screening factor *S*_*ikj*_ is defined as a function of *C*, which is determined as

$$S_{ikj}=f_{c}\left[\frac{C-C_{\min}}{C_{\max}-C_{\min}}\right],$$ (10)

where *C*_max_ and *C*_min_ are the maximum and minimum values of the screening range determined by *C*, respectively. *C* can be calculated as follows:

$$x^{2}+\frac{1}{C}y^{2}={\left(\frac{1}{2}R_{ij}\right)}^{2}.$$ (11)

Here, *x* and *y* are the coordinates of *k* relative to the ellipse, which is determined through the positions of atoms *i*, *j*, and *k*. *C* can be computed from the relative distances among the three atoms, *i*, *j*, and *k*:

$$C=\frac{2\left(X_{ik}+X_{kj}\right)-{\left(X_{ik}-X_{kj}\right)}^{2}-1}{1-{\left(X_{ik}-X_{kj}\right)}^{2}},$$ (12)

where $X_{ik}={\left(\frac{R_{ik}}{R_{ij}}\right)}^{2}$ and $X_{kj}={\left(\frac{R_{kj}}{R_{ij}}\right)}^{2}$ . The Smooth Cutoff Function *f*_c_ is Defined as

$$f_{c}\left(x\right)=\{\begin{array}{cc} 1 & x\geq 1\\ {\left[1-{\left(1-x\right)}^{4}\right]}^{2} & 0<x<1\\ 0 & x\leq 0 \end{array}.$$ (13)

### Determination of the Potential Parameters of the Ti–Cr Binary System

2NN MEAM interaction potential parameters for the Ti, Cr, N, Cr–N and Ti–N systems have already been developed (Baskes, 1992; Kim et al., 2006; Ding and Wang, 2019), as shown in Tables 1, 2. Thus, only the 2NN MEAM potential parameters for the Ti–Cr binary system need to be confirmed. As shown in Table 1, 14 independent parameters are present for each unary system. Among them, *E*_c_, *r*_e_, *α*, and *d* are associated with the state universal equation. Moreover, the parameter *A* appears in the embedding function. The decay lengths ( $\beta^{\left(0\right)}$ , $\beta^{\left(1\right)}$ , $\beta^{\left(2\right)}$ , and $\beta^{\left(3\right)}$ ) and the weight factors ( $t^{\left(1\right)}$ , $t^{\left(2\right)}$ , and $t^{\left(3\right)}$ ) are for the electron density. Additionally, *C*_min_ and *C*_max_ are associated with the many-body screening effect. For each binary system, 13 independent parameters are required besides of the unary parameters: *E*_c_, *r*_e_, *α*, *d*, *C*_min_(*i–i–j*), *C*_min_(*j–j–i*), *C*_min_(*i–j–i*), *C*_min_(*j–i–j*), *C*_max_(*i–i–j*), *C*_max_(*j–j–i*), *C*_max_(*i–j–i*), *C*_max_(*j–i–j*), and *ρ*_0_. These are improved by fitting the alloy system target property, got from experiments or first-principles computing. *E*_c_, *r*_e_, and *α* can be achieved from the experimental data if a stable phase is selected as the reference framework. The atomic electron density scaling factor *ρ*_0_ is the ratio of ${\bar{\rho }}_{B}^{0}$ and ${\bar{\rho }}_{A}^{0}$ (A*–*B = Ti*–*N, Cr*–*N, and Ti*–*Cr).

**TABLE 1:** The 2NN MEAM interaction potential parameters for Ti, Cr and N. The units of the cohesive energy *E*_c_and the equilibrium nearest-neighbor distance *r*_e_ are eV and Å, respectively. All the other parameters are dimensionless. The reference structures for Ti, Cr and N are hcp, fcc and dimer, respectively.

	$E_{c}$	$r_{e}$	α	A	$\beta^{\left(0\right)}$	$\beta^{\left(1\right)}$	$\beta^{\left(2\right)}$	$\beta^{\left(3\right)}$	$t^{\left(1\right)}$	$t^{\left(2\right)}$	$t^{\left(3\right)}$	$C_{\min}$	$C_{\max}$	d
Ti^a^	4.87	2.92	4.63	1.17	1.32	0.0	1.95	5.0	5.3	14.1	-5.0	1.0	1.44	0.0
Cr^b^	4.1	2.495	5.58	0.42	6.81	1.0	1.0	1.0	0.3	5.9	-10.4	0.71	2.8	0.0
N^c^	4.88	1.10	5.96	1.8	2.75	4.0	4.0	4.0	0.05	1.0	0.0	2.0	2.8	0.0

a Ref.(Kim et al., 2006).

b Ref.(Ding and Wang, 2019).

c Ref.(Baskes, 1992).

**TABLE 2:** The 2NN MEAM interaction potential parameters for the binary systems.

Parameter	Ti-N	Cr-N	Ti-Cr
*E*_c_	6.61	5.22	4.605
*r*_e_	2.121	2.074	2.6
*a*	4.829	4.52	4.551
*d*	0.0	0.0	0.0
C_min_ (*i-i-j*)	1.457	1.273	1.20
C_min_ (*j-j-i*)	1.457	1.273	1.20
C_min_ (*i-j-i*)	0.90	0.46	0.49
C_min_ (*j-i-j*)	0.22	0.872	0.26
C_max_ (*i-i*-j)	2.8	2.8	2.8
C_max_ (*j-j-i*)	2.8	2.8	2.8
C_max_ (*i-j-i*)	2.8	2.8	2.8
C_max_ (*j-i-j*)	2.8	2.8	2.8
$\rho_{0}$	18	18	1

For the Ti*–*Cr binary system, in the potential parameters optimization process, the BCC_B2 framework is selected as the reference framework, and the elastic constants are adopted as the target property. Since no information is present regarding the lattice parameter and cohesive energy of B2-type TiCr, the initial values of *E*_c_, *r*_e_, and *α* are obtained *via* first-principles calculation using the Vienna *ab* initio simulation package, VASP (Kresse, 1995; Kresse and Furthmuller, 1996b; Kresse and Furthmuller, 1996a). The parameter *d* for the system is set as the average of *d* for the Ti and Cr unary systems since lacking the necessary data for determining its value. The remaining parameters, four *C*_min_ and four *C*_max_, have a major impact on the characteristics of a binary system. Empirically, all the *C*_max_s can assume appropriate values, which shall be large enough so that the first nearest neighbor of the reference framework is fully unscreened for considerable large thermal vibration. All the *C*_max_s are set to 2.8 herein. Both *C*_min_(Ti–Ti–Cr) and *C*_min_(Cr–Cr–Ti) can be set as the average of *C*_min_(Ti–Ti–Ti) and *C*_min_(Cr–Cr–Cr), obtained in the Ti and Cr unary systems, respectively. Therefore, only the parameters *C*_min_(Ti–Cr–Ti) and *C*_min_(Cr–Ti–Cr) need to be obtained by fitting to the elastic constants of the alloy system. The obtained 2NN MEAM interaction potential parameters of the Ti*–*Cr binary system are presented in Table 2.

### Determination of the Potential Parameters of the Ti*–*Cr*–*N Ternary System

**FIGURE 1:**
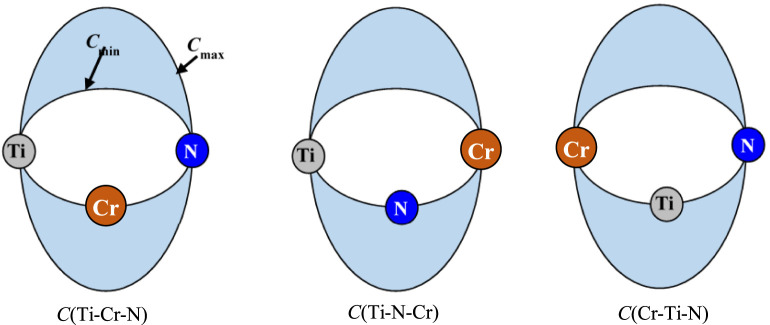
Diagram of the three cases for the interactions screening between two neighboring atoms of various types by a third atom in the Ti–Cr–N ternary system.

To extend the formalism of 2NN MEAM interaction potentials to a ternary system, besides of the parameters of the unary and binary components, three C_min_(*i*–*k*–*j*) and *C*_max_(*i*–*k*–*j*) parameters are required. The C_min_(*i*–*k*–*j*) and *C*_max_(*i*–*k*–*j*) reflect the screening degree of the third atom (*C*) on the interaction between two neighboring *A* and *B* atoms of various types. For the Ti–Cr–N ternary system, the parameters are *C*_min_(Ti–Cr–N), *C*_min_(Ti–N–Cr), *C*_min_(Cr–Ti–N), *C*_max_(Ti–Cr–N), *C*_max_(Ti–N–Cr), and *C*_max_(Cr–Ti–N) (Figure 1).

Due to the difficulty in acquiring enough data to uniquely confirm the ternary PPs, the method for developing the interaction potential parameters of the binary system is generally not applicable for the ternary system. Kim et al. (2009) proposed another approach for obtaining the 2NN MEAM interaction potential parameters of a ternary system in accordance with a type of averaging concept. The method is widely used since it greatly simplifies the optimization process of the interaction potential parameters of a ternary system (Ko and Lee, 2013; Kim et al., 2015; Ding and Wang, 2020). Therefore, the six unknown 2NN MEAM interaction potential parameters of the Ti–Cr–N ternary system can be calculated as follows:

$$C_{min}\left(Ti-Cr-N\right)={\left[0.5{\left(C_{min}^{Ti-Cr-Ti}\right)}^{\frac{1}{2}}+0.5{\left(C_{min}^{N-Cr-N}\right)}^{\frac{1}{2}}\right]}^{2},$$ (14)

$$C_{min}\left(Ti-N-Cr\right)={\left[0.5{\left(C_{min}^{Ti-N-Ti}\right)}^{\frac{1}{2}}+0.5{\left(C_{min}^{Cr-N-Cr}\right)}^{\frac{1}{2}}\right]}^{2},$$ (15)

$$C_{min}\left(Cr-Ti-N\right)={\left[0.5{\left(C_{min}^{Cr-Ti-Cr}\right)}^{\frac{1}{2}}+0.5{\left(C_{min}^{N-Ti-N}\right)}^{\frac{1}{2}}\right]}^{2},$$ (16)

$$C_{max}\left(Ti-Cr-N\right)={\left[0.5{\left(C_{max}^{Ti-Cr-Ti}\right)}^{\frac{1}{2}}+0.5{\left(C_{max}^{N-Cr-N}\right)}^{\frac{1}{2}}\right]}^{2},$$ (17)

$$C_{max}\left(\text{Ti}-\text{N}-\text{Cr}\right)={\left[0.5{\left(C_{\text{max}}^{\text{Ti}-\text{N}-\text{Ti}}\right)}^{\frac{1}{2}}+0.5{\left(C_{\text{max}}^{\text{Cr}-\text{N}-\text{Cr}}\right)}^{\frac{1}{2}}\right]}^{2},$$ (18)

$$C_{max}\left(Cr-Ti-N\right)={\left[0.5{\left(C_{max}^{Cr-Ti-Cr}\right)}^{\frac{1}{2}}+0.5{\left(C_{max}^{N-Ti-N}\right)}^{\frac{1}{2}}\right]}^{2}.$$ (19)

The values of all the interaction potential parameters on the right-hand side of Eqs 14–19 are presented in Table 2. The obtained potential parameters of the Ti–Cr–N ternary system are presented in Table 3.

**TABLE 3:** The 2NN MEAM interaction potential parameters for Ti-Cr-N ternary system.

Parameter	Ti-Cr-N
C_min_(Ti-Cr-N)	0.667
C_min_(Ti-N-Cr)	0.662
C_min_(Cr-Ti-N)	0.24
C_max_(Ti-Cr-N)	2.8
C_max_(Ti-N-Cr)	2.8
C_max_(Cr-Ti-N)	2.8

## Verification of the Interaction Potential

As mentioned in the introduction, the most important validation in the optimization of 2NN MEAM interaction potential parameters is whether the fundamental characteristics of the systems can be reproduced using the obtained interaction potential parameters. For this, the structural, elastic, and surface characteristics of the binary Ti–Cr and ternary Ti–Cr–N systems—which are obtained using the 2NN MEAM interaction potential with the parameters indicated in Tables 2, 3—are compared with the test or other theoretical data. All molecular dynamics simulations in this work are implemented with the January 26, 2017 version of the large-scale atomic/molecular massively parallel simulator package, LAMMPS (Plimpton, 1995). Furthermore, first-principles computing is conducted using VASP to get the physical characteristics of the materials for which the experimental data are not obtained or the theoretical results are less.

### Ti–Cr Binary System

To check the obtained potential parameters reliability for the Ti–Cr binary system, the lattice parameter, cohesive energy, and elastic constants of B2-type TiCr are computed using the newly developed 2NN MEAM potential parameters. Since no information is available regarding these for comparison, their first-principles values are also calculated using VASP modes PW91 and PBE. Table 4 compares the obtained results of the developed MEAM and first-principles. The table shows that the results well agree, except that C_44_ is slightly overestimated by the 2NN MEAM potential parameters.

**TABLE 4:** Comparison of the lattice parameter, cohesive energy and elastic constants of B2-type TiCr calculated by the present developed 2NN MEAM potentials with the first-principles calculation results. The units of the lattice parameter *a,* cohesive energy *E*_c_ and elastic constants are Å, eV and GPa, respectively.

Structure	Property	Present MEAM	First-principles	
			PW91	PBE
B2-type TiCr	*a*	3.02	3.039	3.035
	*E*_*c*_	4.604	4.685	4.685
	C_11_	183.3	204.6	235.9
	C_12_	96.5	80.6	126.6
	C_44_	70.2	41	28.2

To assess the transferability of the developed 2NN MEAM potential parameters, the lattice parameters and cohesive energies of the Laves phase C14-type TiCr_2_, C15-type TiCr_2_, and C15-type Ti_2_Cr are calculated and compared with the test and other calculation results in Table 5. The results of the developed MEAM well agree with the first-principles computing results with an error of about 5%, indicating that the present constructed potentials are suitable for the Ti–Cr alloy system in different frameworks. This result indicates that the newly constructed 2NN MEAM potential parameters are reliable.

**TABLE 5:** Comparison of the present 2NN MEAM lattice parameters and cohesive energies of the Laves phase C14-, C15-type TiCr_2_ and C15-type Ti_2_Cr with other calculation results. The units of the lattice parameter *a* and the cohesive energy *E*_c_ are Å and eV, respectively.

Structure	Property	Present MEAM	Exp	First-principles	
				Present	Previous
C14-type TiCr_2_	*a*	4.857	4.932^a^,4.900^b^	4.859^c^	4.885^d^,4.882^e^
	*c*	7.837	7.961^a^,7.927^b^	7.779^c^	7.830^d^,7.831^e^
	*E*_c_	4.476	-	4.616^c^	4.765^e^
C15-type TiCr_2_	*a*	6.847	6.910^a^	6.854^c^	6.857^d^
	*E*_c_	4.469	-	4.471^c^	-
C15-type Ti_2_Cr	*a*	11.297	-	11.312^c^	-
	*E*_c_	4.698	-	4.968^c^	-

a Ref.(Murray, 1981).

b Ref.(Cuff et al., 1952).

c The first-principles calculation performed in present work.

d Ref.(Chen et al., 2005).

e Ref.(Nong et al., 2013).

Besides, the surface energies of the (001), (110), and (111) surfaces of the B2-type TiCr at 0 K are computed by the established potential parameters. Additionally, the approach put forward by Boettger (1994) is performed to obtain the surface energies:

$$\sigma =\frac{E_{slab}^{N}-N\Delta E}{2A},$$ (20)

where $E_{slab}^{N}$ represents the total energy of an *N*-layer slab, *A* represents the surface area, and ΔE represents the incremental energy impacted by $\left(E_{slab}^{N}-E_{slab}^{N-2}\right)/2$ .

The obtained results are shown in Table 6 and compared to the first-principles computing results. The comparison shows that the results of the developed MEAM are in good agreement with those of the first-principles computing. Moreover, the surface energy orientation dependency is well reproduced. Note that such an agreement is hard to achieve with the previous MEAM potential parameters. Therefore, the newly developed 2NN MEAM potential parameters are reliable.

**TABLE 6:** Comparison of the surface energies of B2-type TiCr at 0 K calculated using the present 2NN MEAM potential with the first-principles calculation results. The unit of the surface energy is J/m^2^.

Surface	Present MEAM	First-principles
(001)	2.389	1.833
(110)	2.486	2.152
(111)	4.180	4.323

### Ti–Cr–N Ternary System

**FIGURE 2:**
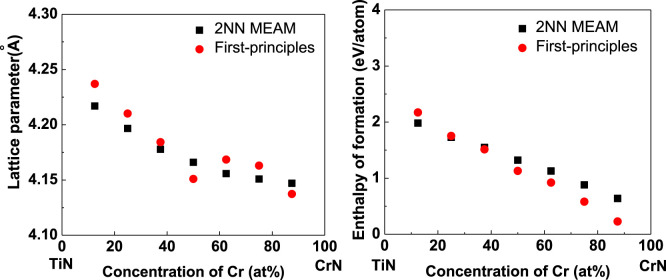
Comparisons of the MEAM and first-principles results for **(A)** the lattice parameter and **(B)** enthalpy of formation of FCC Ti_x_Cr_1-x_N solid solution changing with the atom concentration.

As stated above, the newly developed 2NN MEAM potential parameters of the Ti–Cr binary system can describe the fundamental physical characteristics of the correlative alloys reasonably well. Thus, only the reliability of the potential parameters of the Ti–Cr–N ternary system, which is acquired by combining the already published Ti–N (Ding and Wang, 2019) and Cr–N (Ding and Wang, 2019) potential parameters and the developed Ti–Cr binary potential parameters, needs to be confirmed. Thus, the lattice parameters and enthalpy of formation of the FCC Ti_x_Cr_1-x_N solid solutions with varying atomic concentrations are calculated. In our calculations, the solid solutions (SS) are formulated by substituting part of Ti atoms in a supercell (2 × 1 × 1) of the B1-type TiN with Cr atoms. Since no information is available about these characteristics, the first-principles calculation values are calculated for comparison. The obtained results are compared in Figures 2A,B for lattice parameters and enthalpy of formation, respectively. The figure shows that the results of the newly developed 2NN MEAM conform to first-principles computing results. Moreover, note that the lattice parameters and enthalpy of formation of the FCC Ti_x_Cr_1-x_N SS decrease with increasing Cr atomic concentration, and this trend is also accurately reproduced by the developed 2NN MEAM potential parameters.

To further assess the transferability of the developed potential parameters for the Ti–Cr–N ternary system, the work of adhesion of the TiN/CrN interface are computed and compared to the first-principles computing values (Table 7). The results obtained by the newly developed potential parameters conform to the first-principles computing results, denoting the reliability of the potential parameters of the newly developed Ti–Cr–N ternary system.

**TABLE 7:** The adhesion energies of the TiN/CrN interface calculated by the present ternary potential, comparing with the first-principles calculation values. The unit of the adhesion energy is J/m^2^.

Interface	Present MEAM	First-principles		
		Present^a^	Yin^b^	Chen^c^
(100)	3.839	3.637	3.36	3.47
(110)	5.22	5.328	-	5.79
(111)	8.102	7.679	9.27	-

a The first-principles calculation performed in present work.

b Ref. (Yin et al., 2014).

c Ref. (Chen and Bielawski, 2008).

It has been shown that the developed potential parameters of the Ti–Cr–N ternary system can reasonably accurately reproduce the different fundamental characteristics of the relevant systems. Thus, the developed 2NN MEAM potential parameters can used for performing large-scale atomistic simulations to investigate the enhanced chemical and mechanical characteristics of TiN/CrN multilayered coatings.

## Summary

In this research, the potential parameters for the Ti–Cr binary and the Ti–Cr–N ternary systems on the basis of 2NN MEAM formalism are developed. To verify the dependability of the newly developed potential parameters, the structural, elastic, and surface characteristics of the correlative systems are calculated using the newly developed potential parameters. The developed interatomic potential parameters accurately reproduced the multiple fundamental characteristics of relevant systems conforming to the first-principles calculation and/or experimental results. This study can also contribute to construct the 2NN MEAM potentials parameters of other binary and ternary systems and clarify the underlying mechanism of the hardness enhancement of TiN/CrN multilayered coatings using atomistic simulations.

## Data Availability

The original contributions presented in the study are included in the article/Supplementary Material, further inquiries can be directed to the corresponding authors.
